# Résultat de la radio chimiothérapie concomitante du cancer du col utérin au service oncologie-radiothérapie à l'hôpital universitaire Joseph Ravoahangy Andrianavalona de 2007 à 2009

**DOI:** 10.11604/pamj.2014.19.298.4350

**Published:** 2014-11-18

**Authors:** Ezra Niaina Randriamanovontsoa, Dera Andraina Ratsimandresy, Jean Marc Rakotonarivo, Auberlin Felantsoa Rakototiana, Harinirina Yoël Honora Rantomalala, Florine Rafaramino

**Affiliations:** 1 Service d'Oncologie, CHU Ravoahangy Andrianavalona, Antananarivo 101, Madagascar; 2 Service d'Urologie, HU Ravoahangy Andrianavalona, Antananarivo 101, Madagascar

**Keywords:** Cancer du col, radiothérapie, chimiothérapie, cervical cancer, radiotherapy, chimiotherapy

## Abstract

La radiochimiothérapie devient un standard pour le traitement du cancer du col utérin à partir de IB de mauvais pronostic. L'objectif de ce travail est de rapporter les résultats de cette modalité thérapeutique. Il s'agissait d'une étude rétrospective descriptive des dossiers des patientes atteintes d'un cancer du col utérin du Janvier 2007 au Décembre 2009 traitées par une radiochimiothérapie concomitante. Les patientes ayant reçu une dose inférieure à 45Gy étaient éliminées dans cette étude. Les critères de l'OMS ont été utilisés pour évaluer la réponse tumorale. Au total 46patientes étaient retenues avec un âge moyen de 47ans. Le carcinome épidermoide représentait 89,13%, diagnostiqués au stade localement avancé dans 82,60%. Seulement 26,08% de nos patients ont bénéficié d'une tomodensitométrie abdominale et pelvienne. La dose reçue variait de 45 à 75 Gy. Les résultats thérapeutiques à un mois de la fin du traitement montraient: 45,63% de rémission complète et 30,42% de rémission partielle supérieure à 50%. La toxicité précoce était dominée par la neutropénie chiffrée à 30,55%. A travers de cette étude, la radiochimiothérapie concomitante a amélioré les résultats thérapeutiques à court terme.

## Introduction

Les principes et modalités thérapeutiques des cancers du col utérin sont actuellement bien connus et codifiés. La radiochimiothérapie devient un standard pour le traitement du cancer du col utérin à partir d'un certain stade [1]. Ce travail a pour objectif de rapporter les résultats thérapeutiques du cancer du col utérin traité par l'association radiochimiothérapie concomitante au service d'Oncologie-Radiothérapie de l'hôpital universitaire Joseph Ravoahangy Andrianavalona (CHUA/JRA).

## Méthodes

Il s'agit d'une étude rétrospective descriptive des patientes atteintes de cancer du col utérin vues au service d'Oncologie-Radiothérapie du HU/JRA du 1^er^ Janvier 2007 au 31 Décembre 2009. Nous avons inclus tous les cancers du col utérin prouvés histologiquement et traités par une radiochimiothérapie concomitante. Les patientes qui avaient reçu une dose inférieure à 45Gy étaient exclues. Les paramètres étudiés étaient: les caractéristiques épidémio-cliniques, le stade tumoral clinique, les résultats thérapeutiques à un mois après la fin de l'irradiation. Le protocole de traitement utilisé était le cisplatine 40mg/m^2^, sans dépasser le 70mg, administré de façon hebdomadaire pendant les semaines de radiothérapie. Toutes les patientes étaient traitées par une radiothérapie externe (cobaltothérapie) complétée ou non par une curiethérapie à bas débit. Il consistait à délivrer 1,8 à 2 Gy par jour cinq jours sur sept pendant quatre à six semaines selon la dose totale délivrée. L’évaluation se faisait par un examen gynécologique et par l'imagerie. Les critères de l'OMS ont été utilisés pour évaluer la réponse tumorale.

## Résultats

**Figure 1:**
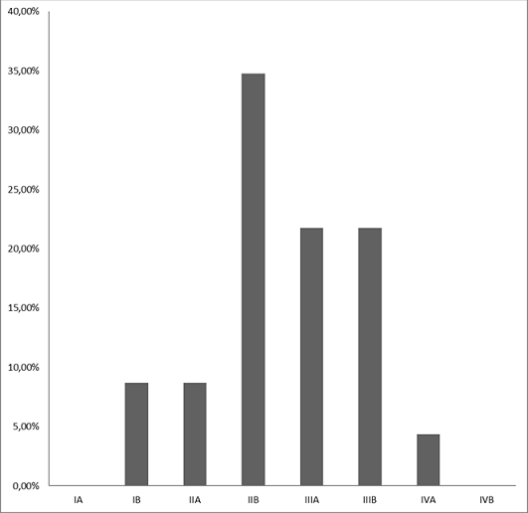
La répartition des patientes selon le stade de FIGO

**Figure 2:**
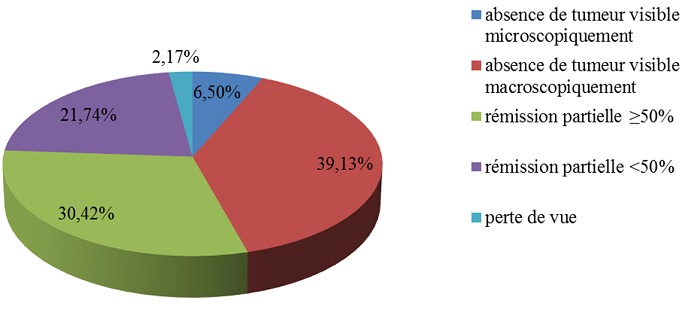
Les réponses tumorales après un mois du traitement

**Figure 3:**
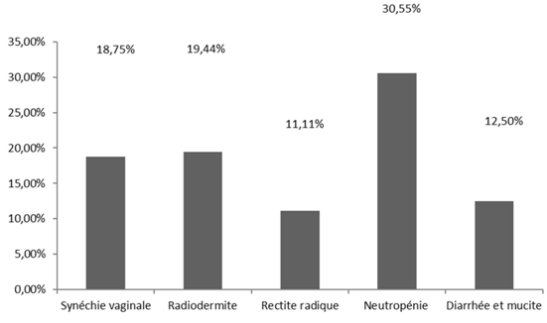
Les effets secondaires du traitement

Pendant ces trois années, 53 dossiers avaient été sélectionnés dont 7 étaient éliminés à cause de la dose reçue par la patiente inférieure à 45 Gy. Au total, nous avons retenu 46 dossiers. L’âge médian des patientes situait à 47 ans avec les deux extrêmes 38 et 71 ans. Elles habitaient à Antananarivo ses périphéries dans 82,60%. La métrorragie représentait 69,56% des circonstances de découverte. Les patientes étaient tabagiques dans 26,09%, multipares dans 82.62%. Le performans statuts de l'OMS variait de 0 à 1 respectivement 80,43% et 19,56%. Le résultat de biopsies du col utérin montrait un carcinome épidermoide dans 89,13%, un adénocarcinome 6,52%, un carcinome à cellule claire et un carcinome adéno-squameux respectivement 2,17%. L'examen gynécologique était réalisé par au moins deux cliniciens, sans anesthésie, avant tout traitement. La Figure 1 représente la stadification selon FIGO. Une radiographie du thorax associée à une échographie ou une tomodensitométrie abdomino-pelvienne étaient demandés respectivement 73,91% et 26,08% à la recherche d'extension locorégionale et à distance. Les doses délivrées variaient de 45 à 75 Gy selon la réponse tumorale (Tableau 1). Les résultats thérapeutiques post irradiation sont visualisés sur la Figure 2. Des effets secondaires, montrés par la Figure 3, étaient observés sur toutes les patientes.

**Tableau 1:** Les doses reçues en Gray (Gy) par les patientes

Dose (Gy)	Nombre	Pourcentage (%)
[45 - 65]	16	34,78
[65 - 75]	30	65,22
Total	46	100

## Discussion

La radiochimiothérapie du col utérin a pu améliorer les résultats thérapeutiques de ce cancer dans le service oncologie. Dans le même service la rémission complète était évaluée à 28,33%, la rémission partielle ≥ 50% à 13,33% et la rémission partielle ≤ 50% à 11,84% dans les années où la radiothérapie associée à la chimiothérapie n’était pas appliquée. NGOMO et al avaient trouvé un résultat comparable: rémission complète à 43,5% [2]. Trois études comparatives ont conclu la supériorité de la radiochimiothérapie concomitante versus radiothérapie seule en termes de réponse tumorale [3–5]. Plus tard cinq autres études randomisées montraient une diminution significative du taux de récidive locorégionale par la radiochimiothérapie concomitante par rapport à la radiothérapie seule. Les auteurs rapportaient aussi que la survie globale et la survie sans récidive sont améliorées significativement par l'administration pendant la radiothérapie d'une chimiothérapie à base de cisplatine [6–9]. Rose et al avaient essayé dans leurs études plusieurs protocoles de chimiothérapies: cisplatine hebdomadaire; cisplatine + 5fluoro-uracile + hydroxyurée; hydroxyurée bihebdomadaire. Ils ont conclu la supériorité des associations contenant du cisplatine par rapport à l'association radiothérapie-hydroxyurée. Du fait de l'existence d'une toxicité plus importante et de l'absence d'un gain thérapeutique de l'association des trois agents chimiques par rapport au cisplatine seul, ils recommandent l'utilisation du cisplatine seul en association à la radiothérapie [6]. C'est pour cette raison que nous avons utilisé le premier type de protocole dans nos patientes. La dose délivrée dépend du stade de la tumeur: 40,8 à 55Gy pour le stade IIB et 51 à 61,2Gy pour les stades III et IVA [7]. L'irradiation peut se faire exclusivement par une radiothérapie externe ou complétée par une curiethérapie [7]. Dans notre étude 78,67% étaient traitées uniquement par une irradiation externe.

Le résultat thérapeutique de ce type d'association provient généralement de l'intrication des effets des deux modalités à plusieurs niveaux [10, 11]. Il existe une synergie ou une potentialisation de l'une à l'autre donnant l'effet thérapeutique supérieur [12, 13]. Le stade du cancer du col est le plus grand facteur pronostique pour le traitement du col utérin. D'après les auteurs de ces quatre études ci-dessus, sans tenir compte de l'envahissement ganglionnaire lombo-aortique, le bénéfice de la radiochimiothérapie concomitante semble moins net pour les stades III et IVA [6–9]. Par contre Peters a trouvé un taux de survie globale à quatre ans de 81% pour les stades IA2, IB, IIA sans envahissement des ganglions lombo-aortiques [14]. Dans notre série 82.60% étaient aux stades localement avancés (IIB à IVA). A la limite de notre bilan d'extension, aucune patiente n'avait présenté une adénopathie profonde. Bien que la radiochimiothérapie concomitante améliore les résultats thérapeutiques, cette association augmente les effets secondaires précoces mais sans différence sur les complications tardives [5]. La neutropénie évaluée à 30,55%, était la plus fréquemment rencontrées dans notre série. Elle était de grade 1 ou 2 et ne nécessitait pas l'arrêt du traitement. La toxicité précoce de la radiochimiothérapie est essentiellement hématologique et digestive [15]. Elle était de grade 3 ou 4 dans 35% pour le groupe combiné, contre 13% pour le groupe radiothérapie seule [16].

## Conclusion

La radiochimiothérapie concomitante a amélioré les résultats thérapeutiques du cancer du col utérin au service d'oncologie CHUA/JRA. La neutropénie représentait la toxicité précoce fréquemment rencontrée. Une étude comparative avec un recul suffisant permettrait de voir l'impact de ce traitement sur la survie des patientes.
